# Oculomotor Nerve Palsy After Influenza Vaccine in Inflammatory Bowel Disease

**DOI:** 10.7759/cureus.3759

**Published:** 2018-12-21

**Authors:** Rajesh Essrani, Rajesh K Essrani, Shehriyar Mehershahi, Arjun K Lohana, Anuraj Sudhakaran

**Affiliations:** 1 Internal Medicine, Lehigh Valley Health Network, Allentown, USA; 2 Internal Medicine, Bronxcare Health System, Bronx, USA; 3 Internal Medicine, Liaquat University of Medical and Health Science, Hyderabad, PAK; 4 Internal Medicine, Geisinger Medical Center, Danville, USA

**Keywords:** inflammatory bowel disease (ibd), influenza, oculomotor nerve palsy

## Abstract

Influenza and pneumococcal vaccines are recommended in inflammatory bowel disease patients. Several neurologic complications have been reported after influenza vaccines, such as Guillain-Barre syndrome, chronic inflammatory demyelinating polyneuropathy, and acute disseminated encephalomyelitis; however, rarely, oculomotor palsy will occur. We report the case of a 23-year-old male with a past medical history of ulcerative colitis on sulfasalazine who presented to the hospital with a complaint of blurry vision five days after an influenza vaccine. Most of the possible causes of oculomotor nerve palsy, such as stroke, intracranial space-occupying lesions, aneurysms, and infections, were ruled out by history, physical exam, blood work, and imaging studies, thus leading to the influenza vaccine as the most likely cause.

## Introduction

The United States Advisory Committee on Immunization Practices (ACIP) recommends an influenza vaccination for all individuals six months of age and older. Patients with inflammatory bowel disease (IBD) are at high risk for infections due to their underlying disease, malnutrition, surgery, or immunosuppressive medications [1-2]. Regardless of immunosuppression, all patients with IBD should be vaccinated for influenza and pneumococcal infection.

Influenza vaccine is very safe and widely recommended to prevent influenza virus infection complications in high-risk populations [3]. It is an inactivated vaccine with a few side effects, such as soreness at the vaccination site, fever, malaise, and myalgia. Several neurologic complications have been reported after influenza vaccines, such as Guillain-Barre syndrome [4], chronic inflammatory demyelinating polyneuropathy [5], acute disseminated encephalomyelitis [6], acute transverse myelitis [7], optic neuritis [8], cerebellar ataxia [9], giant cell arteritis [10], hypoglossal palsy [11], and vasculitis ulnar mononeuropathy, but rarely oculomotor palsy will occur.

We present a rare case report of transient oculomotor palsy after an influenza vaccine in an inflammatory bowel disease patient.

## Case presentation

A 23-year-old male with a past medical history of ulcerative colitis on sulfasalazine presented to the hospital with a complaint of blurry vision five days after an influenza vaccine. He had no medical history of diabetes mellitus, hypertension, glucose intolerance, systemic vasculitis, hyperlipidemia, smoking, or obesity. He denied smoking cigarettes, drinking alcohol, using intravenous drugs, and was sexually active with one female partner only. He had no significant family history of neurological disorders.

On a general physical exam, his blood pressure was 122/67. The ophthalmic exam showed a dilated pupil in the right eye, mild ptosis, and diplopia with reduced adduction, elevation, and depression of the right eye but abduction and intorsion were fine. No abnormality was detected in other cranial nerve exams. Neurological and other system examinations were normal.

On blood work, the complete blood count, comprehensive metabolic panel, erythrocyte sedimentation rate, lipid panel, and hemoglobin a1c (HbA1c) were unremarkable. Imaging of the brain that included computed tomography (CT) of the head without contrast, magnetic resonance imaging (MRI) of the brain with and without intravenous (IV) contrast, and magnetic resonance angiography (MRA) of the head and neck with and without IV contrast were unremarkable. The cerebrospinal fluid analysis was normal. He was treated conservatively with lubricating eye drops, and his symptoms resolved in two weeks.

## Discussion

Influenza vaccine is recommended annually in IBD patients. The influenza vaccine is generally well tolerated with only a few local injection side effects and no documented effects or flare-up of IBD activity [12].

A third cranial nerve palsy can result from lesions anywhere along its path between the oculomotor nucleus in the midbrain and the extraocular muscles within the orbit. It can be complete or incomplete depending on pupillary involvement. Complete nerve palsy presents with an unreactive pupil, paralysis of elevation, adduction, and depression. The eye rests in a position of slight depression, abduction, and intorsion "down and out" [13]. An aneurysm of the posterior communicating artery leads to complete paralysis of cranial nerve (CN) III as parasympathetic ﬁbers run in the periphery of the nerve. Incomplete palsy has pupil sparing, and it is usually caused by nerve ischemia due to hypertension, obesity, and smoking [14-16].

Most of the possible causes, including stroke, intracranial space-occupying lesions, aneurysms, and infections, were ruled out by history, physical examination, blood work, and imaging studies. The fact that this happened immediately after an influenza vaccination makes this a plausible cause, and the rapid improvement of the symptoms in our patient supports the rationale of demyelination for the pathogenesis of transient nerve palsy.

There have been only two case reports of CN III palsy post-influenza vaccine in the literature and only one in an IBD patient [17-18]. Underreporting may be due to the mild and transient nature of the symptoms.

## Conclusions

Influenza is an inactivated vaccine with only a few side effects, like soreness at the vaccination site, fever, malaise, myalgia, and rarely, neurological complications, such as an oculomotor palsy. The ACIP recommends an influenza vaccination for all individuals six months of age and older. It is important to mention that side effects do not outweigh the beneficial effects of the vaccine.
